# Oxaliplatin-dacarbazine combination chemotherapy for the treatment of advanced soft tissue sarcoma of the limbs

**DOI:** 10.1186/1756-9966-28-119

**Published:** 2009-08-26

**Authors:** Xiang-Yun Zong, Yang Yu, Hong-Jian Yang

**Affiliations:** 1 Department of Surgical Oncology, Zhejiang Provincial Cancer Hospital. 38 Guangji Road, Hangzhou, PR China

## Abstract

**Background:**

This study was designed to explore the feasibility, safety, and outcomes of pre-operative oxaliplatin-dacarbazine combination therapy for the treatment of advanced soft tissue sarcoma (STS) of the limb.

**Patients and Methods:**

Between November 2005 and November 2008, 31 patients with advanced limb STS classified with stage IV STS were randomly assigned into experimental or control groups, and both were given 2 cycles of chemotherapy before undergoing surgery. The regimen for the experimental group was oxaliplatin (120 mg/m^2^, d_1_) in combination with dacarbazine (175 mg/m^2^, d_1-3_), while that for the control group was a standard vincristine, epirubicin, cyclophosphamide therapy. Operations were carried out four weeks after the second chemotherapy cycle, followed by another 2-4 more chemotherapy cycles of the previous regimen.

**Results:**

Following preoperative chemotherapy, the experimental group exhibited a significant improvement in tumor regression compared to controls. Both regimens were well-tolerated, and no significant differences in adverse reactions were noted. At a median follow-up of 24 months, 28 patients were still alive and had normal limb function. The progression free survival rate of the experimental group was significantly higher than that of the control group (10/15 vs. 4/16, *p *< 0.05).

**Conclusion:**

Oxaliplatin- dacarbazine neoadjuvant/adjuvant chemotherapy improved the prognosis of patients with advanced limb STS in comparison with vincristine, epirubicin, cyclophosphamide combination therapy.

## Background

Soft Tissue Sarcomas (STS) are malignant tumors that develop within mesenchymal connective tissue and can occur in any part of the body, most commonly in the limbs, which represent over 45% of occurrences [1]. STS growth does not usually cause any noticeable symptoms in early stages, making early detection uncommon. Some STS such as synovial sarcoma, malignant fibrous histiocytoma, rhabdomyosarcoma and certain neurogenic sarcomas tend to invade peripheral tissues, such as nerves, vessels and bones, and are thus have a relatively poor prognosis and are difficult to cure [2].

The treatment of limb STS have traditionally included surgery, which can involve extensive muscle excision or resection [3]. Amputation is not an ideal treatment measure, and is generally opted for only when other treatment has proven unsuccessful [4]. For advanced limb STSs with large tumor mass, distinct local infiltration or post-surgical relapse, chemotherapy or radiotherapy combined with surgery is often the first choice [5-7]. Apart from reducing tumor volume, chemotherapy before surgery can also produce a reaction zone between the tumor and peripheral tissues, which serves as an operational tissue space for surgery. However, it remains unclear whether comprehensive treatment schemes using novel chemotherapy regimens could improve the treatment results and prognoses for advanced limb STS [8]. In the present study, we compared pre-operative chemotherapy with oxaliplatin and dacarbazine to the traditional pre-operative VAC treatment, with the hopes of determining it's safety and to assess whether this regimen imparts a greater advantage, in terms of reducing the tumor margin and increasing progression free survival.

## Patients and Methods

### Inclusion Criteria

① Between 14 years and 70 years of age. ② Female patients that were pregnant or lactating were excluded. ③ No history of chronic primary organ disease, heart failure or other major organ malfunction. ④ The sarcoma originated in limb soft tissue. ⑤ Belong to G1-3T3N0M0 or G1-3T1-3N0-1M1, that is, stage IV according to the Russell GTNM staging system. ⑥ No prior chemotherapy or radiation therapy.

### Patients

Between November 2005 and November 2008, the Department of Surgical Oncology of Zhejiang Provincial Hospital in China received and treated 31 patients with stage IV limb STS. 15 of these were randomly assigned to the experimental group, and the remaining 16 were assigned to the control group. Patients aged between 18 and 66, with a median age of 41 in the experimental group and 50 in the control group (*t *= -0.858, *p *> 0.05). The average tumor size for each group was determined to be in the T3 range (for infiltrating the peripheral vessel, nerve or skeleton). The mean tumor size was 8.4 ± 2.8 cm in the experimental group, and 7.2 ± 1.8 cm (*t *= 1.453, *p *> 0.05). In the experimental group, two patients were diagnosed with regional lymph node metastasis, 2 with lung metastasis. In the control group, 3 patients were diagnosed with regional lymph node metastasis, and 1 with lung metastasis in the control group, the difference in the prevalence of metastases was not significant (χ^2 ^= 0.011, *p *> 0.05). Table 1 shows the clinical characteristics of patients recruited for the study.

**Table 1:** Clinical Characteristics of Patients

		Experimental group (cases)	Control group (cases)
Tumor location	upper arm	3	3
	Thigh	7	11
	lower leg	5	2
Pathological phenotypes	malignant fibrous histiocytoma	8	6
	rhabdomyosarcoma	3	3
	synovial sarcoma	0	4
	malignant nerve sheath tumor	1	1
	clear cell sarcoma	2	0
	unclassifiable	1	2
Cytological grading	G2	0	1
	G3	15	15

The study was conducted according to Good Clinical Practices and was approved by the local ethics committee. All patients gave written informed consent.

### Treatment Regimen

Both groups of patients were examined for contraindications to chemotherapy and surgery. Two cycles of continuous intravenous chemotherapy, 28 days apart, were administered before surgery. For the experimental group, the treatment regimen consisted of 120 mg/m^2 ^d_1 _oxaliplatin (L-OHP) with 175 mg/m^2 ^d_1-3 _dacarbazine (DTIC). The control group received standard VAC chemotherapy 1 mg/m^2^/d_1 _vincristine (VCR), 60 mg/m^2 ^d_1 _epirubicin (Epi-ADM), and 600 mg/m^2 ^d_1 _cyclophosphamide (CTX). Surgical procedures consisting of extensive resection or muscle excision were carried out four weeks after the second cycle, followed by another 2-4 cycles of chemotherapy using the same pre-surgical treatment. Post-operative radiotherapy was undertaken by 3 cases in the experimental group and 10 cases in the control group, respectively.

### Endpoints and adverse reactions

The primary endpoint was progression-free survival, while the secondary endpoints were toxicity of chemotherapy and efficacy of chemotherapy determined by CT or MRI before prior to surgery. Chemotherapeutic response was evaluated using the RECIST criteria. Complete response (CR) was defined as the disappearance of tumors (on the basis of CT scan results) for over 4 weeks, partial response (PR) was defined as the reduction of overall tumor volume by more than 50% for over 4 weeks, and stable disease (SD) was defined as a less than 25% reduction in tumor volume. Chemotherapy toxicity was evaluated in accordance with the CTCAE v3.0 issued by the NCI on August 9, 2006.

### Statistical Analyses

Chemotherapeutic response, surgical margins and therapeutic outcomes were compared between experimental and control groups using Chi-square analyses. Progression free survival time of each group was compared by Log-Rank test. The correlations between chemotherapeutic regimen, chemotherapeutic response, surgical margin and therapeutic outcomes were tested using Pearson's multivariate correlation analyses. All statistical analyses were performed using the SPSS11.5 Software Package.

## Results

**Figure 1:**
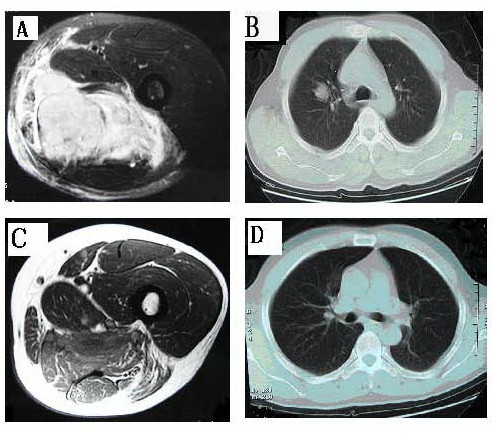
**Image of Typical CR Case**. A. Tumor before chemotherapy. B. Lung metastasis before chemotherapy. C. Tumor after chemotherapy. D. No mass in lung after chemotherapy.

The results from the response evaluation after two cycles of chemotherapy were as follows: 2 CR, 11 PR, and 2 SD in the experimental group; 1 CR, 5 PR, 10 SD in the control group. The difference of response between the two groups was found to be statistically significant (χ^2 ^= 7.878, *p *< 0.05; Table 2). The tumor response rate in the experimental group was 87%, while the tumor response rate in the control group was 38%, correspondingly. Limb-preserving operations were carried out in each case of both groups. But there were 2 cases got positive surgical margin in the experimental group, while 10 cases got positive surgical margin in the control group. Both chemotherapy regimens were well-tolerated with no significant difference between experimental and control group (χ^2 ^= 0, *p *> 0.05). In both groups, no treatment-related deaths occurred, and all adverse reactions were below grade II. Figure 1 shows CT scans of a representative case in the experimental group. Table 3 shows the adverse reactions in detail.

**Table 2:** Statistical Analysis of Therapeutic Response and Prognosis in the Two Groups

		Experimental group (cases)	Control group (cases)	*p *value
Chemotherapy response	CR	2	1	<0.05
	PR	11	5	
	SD	2	10	
Surgical margin	Negative	13	6	<0.01
	Positive	2	10	
Progression free survival	Yes	10	4	<0.05
	No	5	12	

**Table 3:** Adverse Events of Chemotherapy in the Two Groups

AE	Grade (CTCAEv3.0)	Experimental group (cases)	Control group (cases)	*p *value
Nausea	1 (mild)	9	10	>0.05
	2 (moderate)	4	5	
Vomiting	1 (mild)	5	7	>0.05
	2 (moderate)	1	1	
Asthenia	1 (mild)	6	4	>0.05
	2 (moderate)	0	0	
Granulocytopenia	1 (mild)	7	8	>0.05
	2 (moderate)	2	0	
Anaemia	1 (mild)	2	1	>0.05
	2 (moderate)	0	0	
Peripheral Neuropathy	1 (mild)	12	0	Not Comparable
	2 (moderate)	3	0	

**Figure 2:**
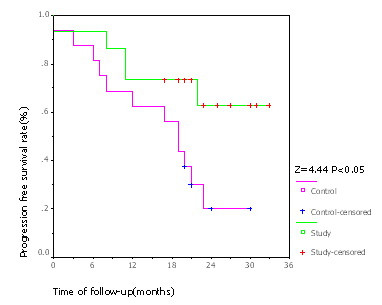
**Kaplan-Meier chart for PFS**. Progression free survival curve showed that PFS of study group was superior to that of control group. "Censored" means cases without endpoint event at the end of follow-up.

**Figure 3:**
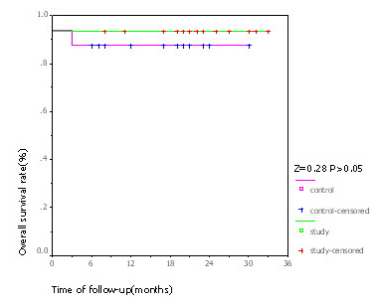
**Kaplan-Meier chart for OS**. Survival curve showed that the difference of OS between the two groups was not significant. "Censored" means cases without endpoint event at the end of follow-up.

At the median follow-up of 24 months, 10 patients were tumor free, sarcoma had relapsed in 4 patients and 1 patient had died in the experimental group. The only death occurred in a patient who did not respond to the chemotherapy and had metastases in both lungs before surgery. In the control group, 4 patients were tumor free, sarcoma persisted in 10 patients, and 2 patients had died. Of the two deaths in the control group, one was found to be with lung metastasis before surgery and died 13 months after operation, the other one suffered from lung metastasis 3 months after operation and died 15 months after operation. The difference of progression free survival between the two groups was significant (χ^2 ^= 5.427, *p *< 0.05; Table 2). Limb functions were essentially normal in all the 28 patients who survived. Median progression-free survival was significantly higher in the experimental group (21 months) compared to the control group (19 months; *Z *= 4.44, *p *< 0.05; Figure 2). Until the end of the follow-up, the difference in overall survival between the two groups was not significant (*Z *= 0.28, *p *> 0.05; Figure 3).

Pearson's multivariate correlation analysis indicated significant correlations between progression free survival (PFS), chemotherapy regimens, chemotherapeutic response, and surgical margin. As shown in Table 4, the treatment given in the experimental group improved chemotherapeutic response, reduced the positive rate of the surgical margin and increased PFS, chemotherapeutic response had a correlation with negative surgical margin and PFS, and negative surgical margin had a increased PFS.

**Table 4:** Multivariate Correlation Analysis

		Chemotherapy response	Surgical margin	Tumor-free survival
Chemotherapy Regimen	Pearson correlation	0.484	0.504	0.418
	Sig. (2-tailed)	<0.01	<0.01	<0.05
Chemotherapy response	Pearson correlation		0.965	0.683
	Sig. (2-tailed)		<0.001	<0.001
Surgical margin	Pearson correlation			0.721
	Sig. (2-tailed)			<0.001

## Discussion

In this study, a combination of oxaliplatin-dacarbazine was used as neoadjuvant/adjuvant chemotherapy, with the intention of exploring the usefulness of this regimen as a safe and effective treatment for advanced limb STS. This combination chemotherapy was generally well tolerated and no serious adverse events were noted during or after chemotherapy. Compared to a traditional VAC regimen, oxaliplatin-based chemotherapy significantly improved prognosis over the median follow-up duration of 24 months and improved the negative rate of surgical margin to a greater degree in patients with stage IV limb STS. Importantly, oxaliplatin combination therapy significantly increased progression free survival over the study period. These results indicate that oxaliplatin-dacarbazine chemotherapy can effectively improve tumor remission in patients with advanced limb STS compared to traditional VAC scheme.

### Safety of the Oxaliplatin-Dacarbazine Treatment

In this study, we used a combination of oxaliplatin and dacarbazine as neoadjuvant/adjuvant chemotherapy to determine the safety and efficacy of this treatment for advanced limb STS. To our knowledge, this study constitutes the first report for the use of oxaliplatin in the treatment of advanced STS. Previously, oxaliplatin has been used to treat malignant tumors in the digestive system, ovarian cancer, breast cancer, lymphoma, small cell lung cancer, among others and its safety has been widely confirmed. A phase I and pharmacokinetic study of pemetrexed in combination with oxaliplatin was ever performed to determine the maximum tolerated dose (MTD), and to evaluate safety and pharmacokinetics in patients with metastatic solid tumors. Thirty-six patients with advanced tumors were observed, including 5 patients with sarcomas. This study demonstrated that the combination of pemetrexed plus oxaliplatin is feasible and can be safely administered every 21 days in patients with solid tumors. Toxic effects were predictable, reversible and manageable, with neutropenia being the primary toxicity and no unexpected toxicity observed. The recommended dosage for oxaliplatin was 120 mg/m^2 ^[9]. Dacarbazine is considered a critical chemotherapeutic agent in comprehensive treatment regimes for advanced STSs [10,11]. Patients in both the experimental and control groups experienced grade 1 to 2 adverse effects, consisting mainly of digestive and blood system toxicity. All patients had mild to moderate peripheral neuropathy, which remitted following the drug treatment, as expected from previous studies. No severe adverse reactions occured in the experimental group suggesting that oxaliplatin-dacarbazine combination treatment is likely to be tolerable and safe for patients with limb STS.

### Oxaliplatin-based adjuvant chemotherapy for the treatment of advanced limb STS

Despite the small sample size of this study, our results show a clear advantage in the use of oxaliplatin-based neoadjuvant chemotherapy: the tumor response rate in the experimental group was 87%, limb-preserving operations were carried out in all cases. In addition, this combination therapy may also prove beneficial for the treating of distant metastatic tumors, this hypothesis is supported by the fact that one patient's lung metastasis disappeared after the first cycle of chemotherapy.

Our follow-up analysis at a median of 24 months revealed that all patients from the experimental group who showed significant benefits of chemotherapy before surgery were still alive, including survivors with and without tumors. The only death occurred in a patient who did not respond to the chemotherapy and had metastases in both lungs before surgery. In general, the prognoses for patients with distant metastases were much worse, with a shorter progression-free stage. Prognoses were best for patients who had no distant metastasis before surgery and who showed significant chemotherapeutic response, this was similar to observations seen in another study [12]. Patients in the experimental group mainly benefited from tumor-free survival, without a corresponding increase in overall survival. There was no significant difference in overall survival time between experimental and control groups, which may reflect the short follow-up time and the small sample size of the study. Future studies using larger cohorts and a longer follow-up time may reveal survival benefits. In addition, we discovered that the two CR cases from the experimental group were both patients with neurogenic tumors. Whether neurogenic tumors are more sensitive to oxaliplatin-dacarbazine treatment is worthy of further investigation [13].

## Competing interests

The authors declare that they have no competing interests.

## Authors' contributions

XYZ conceived the study, carried out all experiments and drafted the manuscript. YY and HJY participated in the study design and revised the manuscript.

## References

[B1] Brennan MF (2005). Soft tissue sarcoma: advances in understanding and management. Surgeon.

[B2] Leidinger B, Heyse T, Schuck A, Buerger H, Mommsen P, Bruening T, Fuchs S, Gosheger G (2006). High incidence of metastatic disease in primary high grade and large extremity soft tissue sarcomas treated without chemotherapy. BMC Cancer.

[B3] Stoeckle E, Gardet H, Coindre JM, Kantor G, Bonichon F, Milbéo Y, Thomas L, Avril A, Bui BN (2006). Prospective evaluation of quality of surgery in soft tissue sarcoma. Eur J Surg Oncol.

[B4] Anacak Y, Sabah D, Demirci S, Kamer S (2007). Intraoperative extracorporeal irradiation and re-implantation of involved bone for the treatment of musculoskeletal tumors. J Exp Clin Cancer Res.

[B5] Thijssens KM, Hoekstra-Weebers JE, van Ginkel RJ, Hoekstra HJ (2006). Quality of life after hyperthermic isolated limb perfusion for locally advanced extremity soft tissue sarcoma. Ann Surg Oncol.

[B6] Kraybill WG, Harris J, Spiro IJ, Ettinger DS, DeLaney TF, Blum RH, Lucas DR, Harmon DC, Letson GD, Eisenberg B (2006). Radiation Therapy Oncology Group Trial 9514: Phase II study of neoadjuvant chemotherapy and radiation therapy in the management of high-risk, high-grade, soft tissue sarcomas of the extremities and body wall: Radiation Therapy Oncology Group Trial 9514. J Clin Oncol.

[B7] Grunhagen DJ, de Wilt JH, Graveland WJ, Verhoef C, van Geel AN, Eggermont AM (2006). Outcome and prognostic factor analysis of 217 consecutive isolated limb perfusions with tumor necrosis factor-alpha and melphalan for limb-threatening soft tissue sarcoma. Cancer.

[B8] Bauer S, Hartmann JT (2006). Locally advanced and metastatic sarcoma (adult type) including gastrointestinal stromal tumors. Crit Rev Oncol Hematol.

[B9] Misset JL, Gamelin E, Campone M, Delaloge S, Latz JE, Bozec L, Fumoleau P (2004). Phase I and pharmacokinetic study of the multitargeted antifolate pemetrexed in combination with oxaliplatin in patients with advanced solid tumors. Ann Oncol.

[B10] Verma S, Younus J, Stys-Norman D, Haynes AE, Blackstein M (2007). Ifosfamide-based combination chemotherapy in advanced soft-tissue sarcoma: a practice guideline. Curr Oncol.

[B11] Kopp HG, Patel S, Brücher B, Hartmann JT (2008). Potential combination chemotherapy approaches for advanced adult-type soft-tissue sarcoma. Am J Clin Dermatol.

[B12] Meza JL, Anderson J, Pappo AS, Meyer WH, Children's Oncology Group (2006). Analysis of prognostic factors in patients with nonmetastatic rhabdomyosarcoma treated on intergroup rhabdomyosarcoma studies III and IV: the Children's Oncology Group. J Clin Oncol.

[B13] Carli M, Ferrari A, Mattke A, Zanetti I, Casanova M, Bisogno G, Cecchetto G, Alaggio R, De Sio L, Koscielniak E, Sotti G, Treuner J (2005). Pediatric malignant peripheral nerve sheath tumor: the Italian and German soft tissue sarcoma cooperative group. J Clin Oncol.

